# Transposon Removal Reveals Their Adaptive Fitness Contribution

**DOI:** 10.1093/gbe/evae010

**Published:** 2024-01-20

**Authors:** Susanne Cranz-Mileva, Eve Reilly, Noor Chalhoub, Rohan Patel, Tania Atanassova, Weihuan Cao, Christopher Ellison, Mikel Zaratiegui

**Affiliations:** Department of Molecular Biology and Biochemistry, Rutgers, the State University of New Jersey, Piscataway, NJ, USA; Department of Molecular Biology and Biochemistry, Rutgers, the State University of New Jersey, Piscataway, NJ, USA; Department of Molecular Biology and Biochemistry, Rutgers, the State University of New Jersey, Piscataway, NJ, USA; Department of Molecular Biology and Biochemistry, Rutgers, the State University of New Jersey, Piscataway, NJ, USA; Department of Molecular Biology and Biochemistry, Rutgers, the State University of New Jersey, Piscataway, NJ, USA; Department of Genetics, Rutgers, the State University of New Jersey, Piscataway, NJ, USA; Department of Genetics, Rutgers, the State University of New Jersey, Piscataway, NJ, USA; Department of Molecular Biology and Biochemistry, Rutgers, the State University of New Jersey, Piscataway, NJ, USA

**Keywords:** transposons, evolution, fitness

## Abstract

Transposable elements are molecular parasites that persist in their host genome by generating new copies to outpace natural selection. Transposable elements exert a large influence on host genome evolution, in some cases providing adaptive changes. Here we measure the fitness effect of the transposable element insertions in the fission yeast *Schizosaccharomyces pombe* type strain by removing all insertions of its only native transposable element family, the long terminal repeat retrotransposon Tf2. We show that Tf2 elements provide a positive fitness contribution to its host. Tf2 ablation results in changes to the regulation of a mitochondrial gene and, consistently, the fitness effect are sensitive to growth conditions. We propose that Tf2 influences host fitness in a directed manner by dynamically rewiring the transcriptional response to metabolic stress.

**Significance**

Transposable elements (TEs) are generally considered molecular parasites that can occasionally provide adaptive mutations to their host. In fungi, TEs have evolved strategies to minimize their fitness burden, and previous studies in fission yeast showed that the individual Tf2 insertions present do not affect fitness. We have removed all the Tf2 copies in the laboratory strain of fission yeast, observing that their collective fitness contribution is positive. The preference of Tf2 to insert near stress-regulated genes indicates that this could constitute a strategy for the TE to maintain its presence in the host genome.

## Introduction

Transposable elements (TE) are virtually ubiquitous molecular parasites. Their overwhelming success in exploiting cellular processes is commensurate with the influence they exert on the genomic evolution of their hosts (Burt and Trivers 2006; Wells and Feschotte 2020). The question of how TE expands and wanes within a host population is far from clear due to the extremely complex interplay of TE mobility, host defense mechanisms, and host population genetics (Charlesworth et al. 1994). TE is generally thought to impose a negative fitness effect (Eanes et al. 1988; Houle and Nuzhdin 2004; Pasyukova et al. 2004; Baduel et al. 2021), but examples of adaptive changes caused by TE activity abound (Quadrana et al. 2016, 2019; Cosby et al. 2019; Rech et al. 2019). Whether TE-induced adaptive mutations are a significant contributor to host evolution, or simply restricted to happenstance generation of “hopeful monsters” (Erwin and Valentine 1984) remains an open question.

Experimental approaches to investigate the influence of TE on host fitness use model organisms with established and active TE colonies. These experiments generally follow the expansion of the TE insertions in a susceptible population in conditions of relaxed selection and then evaluate the fitness of new insertions or TE overaccumulation. Efforts in the fly *Drosphila melanogaster* (Eanes et al. 1988), the plant *Arabidopsis thaliana* (Quadrana et al. 2019), and yeasts *Saccharomyces cerevisiae* (Wilke and Adams 1992; Scheifele et al. 2009) and *Schizosaccharomyces pombe* (Esnault et al. 2019) revealed that new insertions are on average neutral to mildly deleterious, and very rarely provide adaptive value to the host. However, overall transposon dosage sometimes shows a cumulative or synergic negative effect likely caused by increased ectopic recombination between dispersed TE copies, leading to sterility, structural mutations with large effects, and increased replicative stress (Lee 2022).

Such experiments can however only measure the effect of new insertions but fail to consider that of copies fixed in the experimental population. Comparative genomics surveys of wild isolates can reveal information about the distribution of TE but their analysis is complicated by the additional genetic variation involved, making it difficult to estimate the relative contribution of selective and neutral processes in the population dynamics of TE. Therefore, the question of whether TE can achieve equilibrium densities or is always involved in boom and bust cycles remains unsolved.

The fission yeast *S. pombe* is an excellent model organism for TE research. All laboratory strains are derived from a single isolate whose genome has been completely sequenced (Hoffman et al. 2015). This strain is colonized by a single family of TE, the gypsy-type long terminal repeat (LTR) retrotransposon Tf2, present in 13 full-length copies in the sequenced genome (Bowen et al. 2003). An additional highly related TE, Tf1, can be found in other fission yeast isolates (Esnault and Levin 2015). As is often the case for TE found in genomes with high gene density, Tf1 and Tf2 have evolved insertion target site selection mechanisms to minimize their mutagenic potential, in this case through interactions between the integrase and the host DNA binding factor Sap1 that guide insertion to promoters of protein-coding genes (Hickey et al. 2015; Jacobs et al. 2015). Owing to this preference, it has been proposed that Tf1/2 TE can contribute adaptive value to their host by rapidly rewiring the transcriptional response to stressors that often also activate TE expression. Indeed, adaptive insertions that enable survival to CoCl_2_ treatment can be selected from a library of Tf1 transpositions (Esnault et al. 2019). These insertions upregulate transcription of stress response genes driven by the targeted promoters (Feng et al. 2013). A potential role of Tf1/2 elements in adaptive evolution of regulatory sequences is supported by the observation that polymorphic Tf1/2 insertions in a collection of natural fission yeast isolates are enriched in genes involved in sporulation, a common response to natural stressors (Esnault et al. 2019). Adaptive contributions to host fitness may explain the persistence of Tf1/2 elements. But population genetics surveys of Tf1 and Tf2 indicate that a recent hybridization event led to a burst of transposition that is subject to negative selection, leading to a high diversity of polymorphic insertions, with only one single example of a fixed insertion (Tusso et al. 2022). A recent study directly tested the fitness effect of each of the 13 individual insertions present in the type strain, finding that they are neutral (Wang et al. 2021). However, this study did not remove the insertions, but instead substituted them with an antibiotic resistance marker. In summary, there is inconclusive experimental support for adaptive, neutral, or maladaptive effects of Tf1/2 TE on the fission yeast genome.

Dissecting the complex relationships between TE and their hosts will require experiments that directly test these hypotheses in completely controlled conditions. Such approaches would benefit from the availability of a host where all the original TE has been removed, providing a *tabula rasa* experimental platform. Previous efforts fully removed the Ty1 TE from *S. cerevisiae* and *Saccharomyces paradoxus* backgrounds (Wilke and Adams 1992; Garfinkel et al. 2003; Ahn et al. 2017; Chen et al. 2022), enabling direct investigations of various aspects of Ty1 biology. Experiments that evaluate the change in fitness resulting from the loss of insertions present in the host genome may inform models for the evolution of host-TE interaction. Here, we develop a fission yeast background completely free of TE, through removal of all Tf2 insertions present in the type strain through inter-LTR recombination, the main process that deletes LTR TE in a natural setting, and determine their effect on host fitness.

## Results

### Development of TE-Free Fission Yeast Strains

**Fig. 1.**
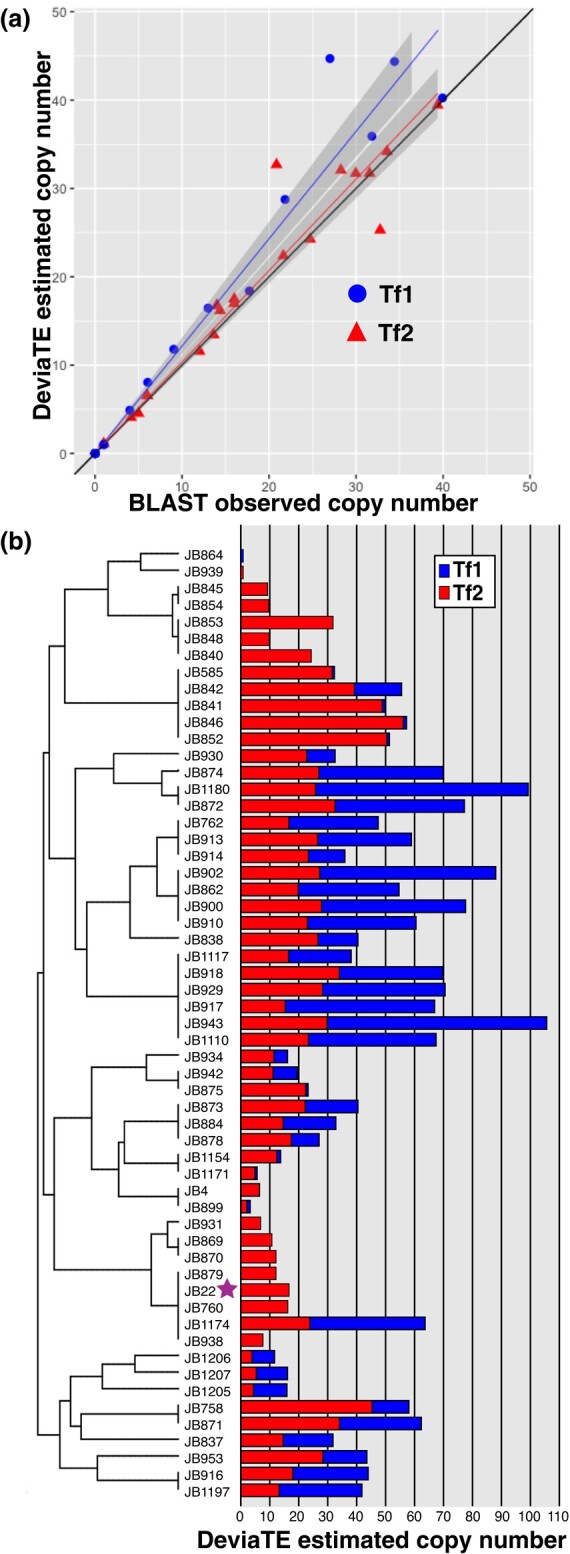
a) Scatterplot of Tf1 and Tf2 copy number estimated by DeviaTE analysis of short read sequencing data (Jeffares et al. 2015) (*y-*axis) and observed copy number as measured by BLAST alignment on long-read assemblies of the same strains (Tusso et al. 2019) (*x-*axis). b) Estimated copy number of Tf1 and Tf2 in natural isolates arranged in the phylogeny tree described in Jeffares et al. (2015). A purple star depicts the commonly used 972 laboratory strain.

In order to directly evaluate the effect of the Tf2 insertions on the fission yeast fitness, we sought to remove all copies present, with the goal of comparing isogenic strains differing only in the presence of TE. First, we evaluated the number of copies of Tf1 and Tf2 present in a panel of natural isolates of fission yeast (Jeffares et al. 2015), using the available long-read sequencing assemblies (Tusso et al. 2019) to calibrate copy number estimates from short-read sequencing data via the DeviaTE analysis pipeline (Weilguny and Kofler 2019) (Fig. 1). The results revealed a wide variability in Tf1 and Tf2 copy numbers. The laboratory-type strain isolate (972/JB22) is present in a clade where Tf1 has become extinct, and in comparison with other isolates presents a moderate number of Tf2 copies of 13 to 15 copies per genome (Bowen et al. 2003; Wang et al. 2021; Tusso et al. 2022). Laboratory derivatives of 972 showed some polymorphism in Tf2 copy number. In particular, the Tf2-7/8 array, consisting of 972 of 2 tandemly arranged copies sharing a central LTR, showed 3 tandem copies in strains CHP428/CHP429 and 4 tandem copies in strain PB1. Additionally, strains CHP428/429 exhibited a novel Tf2 insertion not present in the original 968/972/974 isolates which we named Tf2-14 (genome coordinates I:4940032). Despite the fact that it is not the strain with the smallest number of Tf1/2 insertions we decided to remove all Tf2 copies in 972 strain derivatives so as to take advantage of the thoroughly characterized physiology of this isolate in subsequent characterization.

LTR retrotransposons are inherently unstable due to frequent intrachromatid recombination (ICR) between the flanking LTR that results in loss of the coding sequence (CDS), leaving a solo LTR (Roeder and Fink 1980; Winston et al. 1984; Aguilera et al. 2000; Mieczkowski et al. 2006). We sought to take advantage of this phenomenon to remove the Tf2 CDS. We first attempted to remove Tf2 copies by CRISPR/Cas9 mediated cleavage of the Tf2 CDS (Jacobs et al. 2014). We reasoned that cleavage would destabilize the insertion, engaging Homologous Recombination, and provide a negative selection for the presence of the CDS that would enable us to isolate inter-LTR recombination events (supplementary fig. S1a and b, Supplementary Material online). This strategy yielded 2 independent strains showing complete loss of Tf2 CDS after 4 rounds of CRISPR plasmid transformation and removal (supplementary fig. S1c, Supplementary Material online). However, these strains exhibited very poor spore viability upon backcross with the original strain, but not in a selfing cross, suggesting that they had acquired chromosomal rearrangements (Qi et al. 2023). Indeed, long-read high throughput sequencing revealed multiple rearrangements, including pericentric and paracentric inversions, balanced translocations, and deletions. The breakpoints of these rearrangements were the Tf2 insertions targeted by CRISPR (supplementary fig. S1d and e, Supplementary Material online). Characterization of the partially deleted intermediate strains revealed that the rearrangements could occur without deletion of the CDS, in a transformation round previous to the one that caused deletion by inter-LTR recombination. Thus, while CRISPR/Cas9 cleavage does provide a rapid method to select for inter-LTR recombination, it also leads to nonallelic recombination between the interspersed Tf2 insertions.

**Fig. 2.**
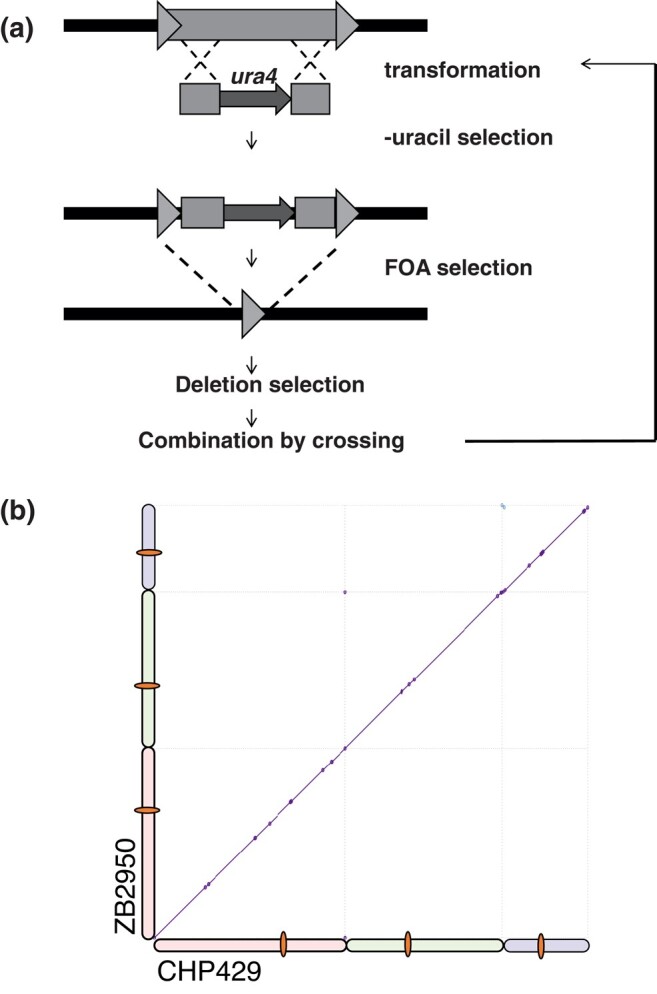
a) Tf2 removal scheme by *ura4* tagging and 5FOA selection of deletion recombinations. b) Full genome alignment of a parental strain (CHP429) and a Tf-null derivative (ZB2950).

To avoid nonallelic recombinations in the deletion process, we undertook a classic recombineering approach to remove Tf2 CDS. LTR TE can be deleted by placing a negatively selectable marker within its CDS and then counterselecting against the marker. This method allowed measurement of inter-LTR recombination rates in Ty elements soon after their initial discovery (Winston et al. 1984), and has been used to remove entire families of Ty elements from *S. cerevisiae* (Ahn et al. 2017). By transformation of a linear DNA fragment consisting of the *ura4* gene flanked by Tf2 CDS homology arms, we were able to randomly tag individual Tf2 insertions through selection in media without uracil. Subsequent selection in 5FOA media yields strains where the *ura4* gene has been lost either by gene conversion, reverting the insertion to its native state, or by inter-LTR recombination, which removes the CDS leaving a solo LTR (Fig. 2a). We then selected strains with deleted individual Tf2 CDS and combined the deletions by crossing. This process was repeated until all Tf2 CDS were removed (supplementary fig. S2a and b, Supplementary Material online). We performed long and short read whole genome sequencing to fully characterize the genome of the original parental strains and the Tf2-free derivatives. The assembled genomes confirmed the complete removal of all Tf2 coding sequences with no rearrangements or deletions (Fig. 2*b* and supplementary fig. S2c, Supplementary Material online). We subsequently refer to these strains at the Tf-null background.

The Tf-null strains obtained from the Tf2-CDS removal process had only acquired 4 single nucleotide polymorphisms, one of which was a nonsynonymous mutation located in a nonconserved region of the protein-coding gene *cys12* (*cys12-A13S*) (supplementary table S1, Supplementary Material online) (Fujita and Takegawa 2004; Sideri et al. 2015). In order to account for the fitness effect of this mutation in the analysis of the Tf-null strains, we introduced it via CRISPR/Cas9-directed mutagenesis into a wild type (WT) background. By competitive growth assays we measured the fitness of 4 independent cys12-A13S mutants relative to the parent strain, to account for potential off-target effects of the CRISPR manipulation. The 4 isolated mutants showed a spread of small positive and negative relative fitnesses (supplementary fig. S3, Supplementary Material online), indicating that the *cys12-A13S* mutation does not confer a defined fitness effect to the Tf-null background.

### Tf2 Insertions Have a Positive Fitness Effect

**Fig. 3.**
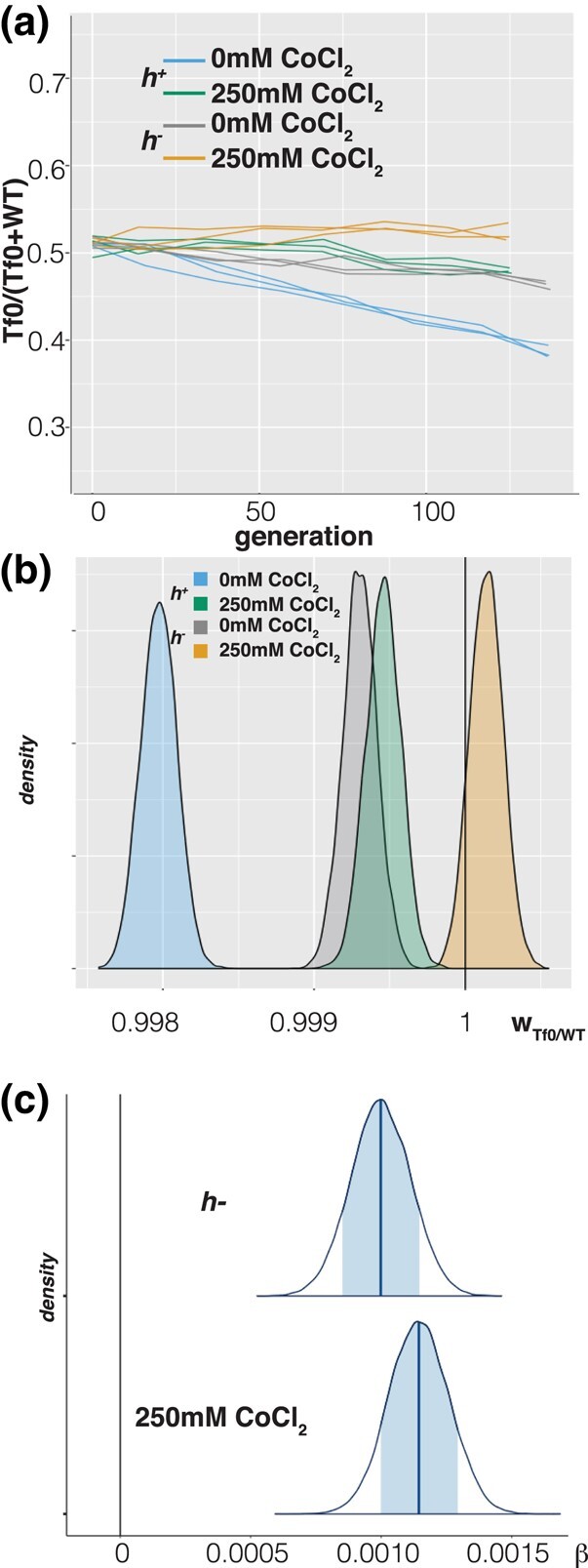
WT versus Tf-null competitive growth assays. a) Frequency of the Tf-null specific polymorphism in competition cultures of both mating types (h^+^/h^−^) with and without treatment with 250 μM CoCl_2_. b) Density plots of posterior probability distributions of relative fitness of Tf-null over WT (w_Tf0/WT_). The vertical line at 1 depicts the expectation of neutrality. c) Density plots of posterior probability distribution of the coefficients for h^−^ and CoCl_2_ treatment. The vertical line at 0 depicts the expectation of no effect of the factor on w_Tf0/WT_.

The availability of isogenic strains differing only in the presence or absence of TE allows for the direct measurement of the effect of the TE insertions on host fitness. We carried out a competition experiment to measure this effect. In addition, we evaluated the effect of treatment with CoCl_2_, which mimics hypoxic conditions and activates Tf2 transcription (Esnault et al. 2019), as well as the effect of mating type to account for the potential effect of expression in h^−^ cells of *mat1-m* encoded factor Mc, a homolog of the sex determination factor SRY (Sex-determining Region Y) that binds to LTR (Matsuda et al. 2011). For this experiment, we mixed equal numbers of cells of the same mating type to prevent sexual reproduction, and passaged them twice a day for ∼140 generations, ensuring the cultures never reached saturation (Fig. 3a). We then fit the genotype proportions as a function of relative fitness between the 2 genotypes, with additive effects of mating type and CoCl_2_ treatment, and number of generations (Kronholm et al. 2020). If the presence of the Tf2 insertions confers a negative fitness effect on the host we would observe a relative fitness w_tf0/wt_ > 1; if the Tf2 insertions brings adaptive value to the host we would observe w_tf0/wt_ < 1. Surprisingly the baseline w_tf0/wt_ is 0.9981; [0.9980, 0.9983] (median, [highest posterior density interval (HPDI) 89%]) (Fig. 3b), indicating that the Tf2 insertions confer a fitness advantage. The total selection coefficient *s*_Tf2_ is −2e−3, and assuming a purely additive effect of all Tf2 insertions the average $\bar{s}$_Tf2_ is −1.5e−4 per insertion. Both the h^−^ mating type (0.001; [0.0008,0.0012]:β_mat_ median, [HPDI 89%]) and CoCl_2_ treatment (0.001; [0.0009,0.0013]:β_CoCl2_ median; [HPDI 89%]) influenced w_tf0/wt_ with a positive sign (Fig. 3c) indicating that Mat2Mc expression and CoCl_2_ treatment decreased the beneficial effect of Tf2 presence on fitness. To account for the possible influence of the Cys12A13S mutation present in the Tf-null strains, we re-analyzed the competition assay data to directly compare the relative fitness of the *c*y*s12*-*A13S* mutants with that of the Tf-null strains, estimating the effect of lacking the Tf2 insertions as a coefficient influencing relative fitness. The estimated effect of lacking the Tf2 insertions (β_Tf-null_ = −0.0039; [−0.0041, −0.0037]:median, [HDPI 89%]) confirmed the beneficial effect of Tf2 insertions on fitness.

### Tf2 Insertions Target ncRNA Stress Regulons

**Fig. 4.**
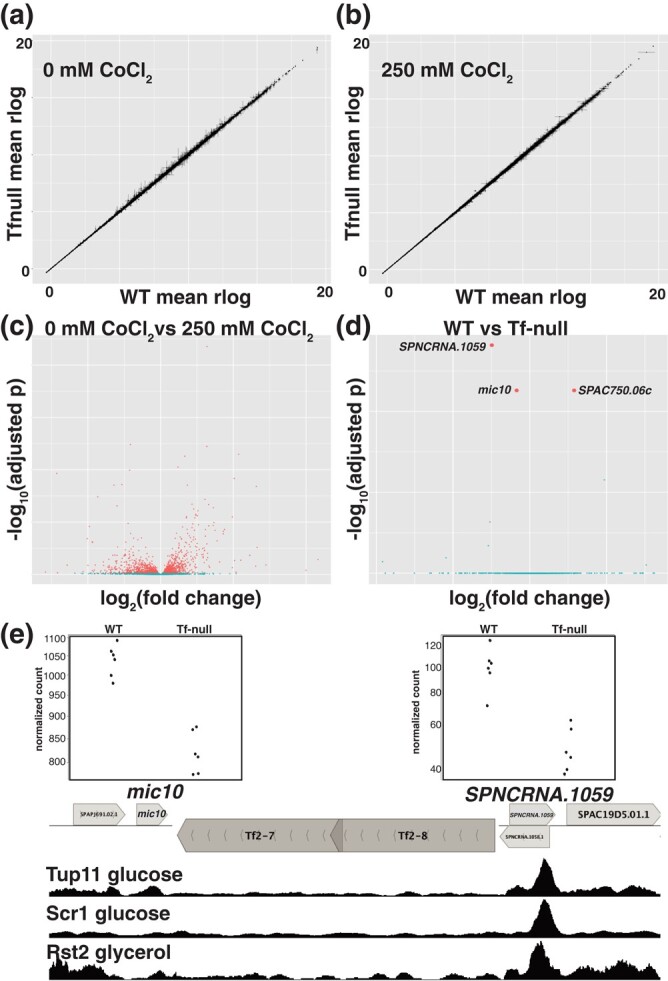
Influence of the Tf2 insertions on host gene expression. a, b) Scatterplot of mean regularized log (rlog) transformed RNAseq read counts of WT and Tf-null strains in a) 0 μM and b) 250 μM CoCl_2_. c, d) Volcano plots of c) 0 mM versus 250 μM CoCl_2_ and d) WT versus Tf-null strains RNAseq. The 3 genes detected as significantly changed in the Tf-null strain are labeled by name. e) Genomic map of the 2 genes changed in Tf-null, positioned adjacent to the Tf2-7/8 array, with normalized read counts (above) and Tup11, Scr1, and Rst2 enrichment (from Vassiliadis et al. 2019) below.

The related TE Tf1 has shown the potential to provide adaptive value by influencing the expression of stress response genes (Feng et al. 2013; Esnault et al. 2019). To investigate the mechanism by which the Tf2 insertions increase the fitness of the host genome and CoCl_2_ treatment decreases fitness in the presence of Tf2 we performed RNAseq in WT and Tf-null cells grown with and without CoCl_2_, with a factorial design to measure main effects and interaction. While CoCl_2_ treatment results in widespread expression differences (Fig. 4a and c), the absence of Tf2 elements had virtually no effect on gene expression in either condition (no interaction between CoCl_2_/Tf-null detected, Fig. 4b and d). Only 3 genes showed differential expression between the WT and Tf0 strains (Fig. 4d). One of these, SPAC750.06c, is located in the subtelomeric region, which is repetitive and often rearranged leading to artifactual results in RNA-seq experiments (Oizumi et al. 2021), so we did not characterize it further. The 2 other differentially expressed genes, *mic10* and the noncoding RNA (ncRNA) *SPNCRNA.1059*, immediately flank the Tf2-7/8 array insertion (Fig. 4e). Mic10 is a subunit of the mitochondrial contact site and cristae organizing system complex, an organizer of the inner mitochondrial membrane, where it enables respiratory metabolism (Malecki and Bähler 2016; van der Laan et al. 2016). In fission yeast, many respiratory metabolism genes are regulated in *cis* by expression of nearby ncRNA (Hirota et al. 2008; Oda et al. 2015). The transcriptional regulation that enables the shift from fermentative to respiratory metabolism upon diauxic shift is mediated by the coordinated action of DNA binding factors Scr1, Tup11, and Rst2 (Vassiliadis et al. 2019). We plotted the binding of Scr1 and Tup11 in cells grown in glucose rich media as well as Rst2 in cells starved for glucose and observed clear peaks for these factors downstream of *SPNCRNA.1059* (Fig. 4e). Consistently, both *SPNCRNA.1059* and *SPNCRNA.1058*, an overlapping ncRNA antisense to *mic10*, become upregulated in glucose and sucrose starvation conditions (Vassiliadis et al. 2019). This arrangement is typical for a regulatory cassette that responds to the diauxic shift, suggesting that the insertion of Tf2-7/8 and subsequent amplification of the array to 2 or more tandem copies separated *mic10* from its *cis* ncRNA transcriptional regulators.

**Fig. 5.**
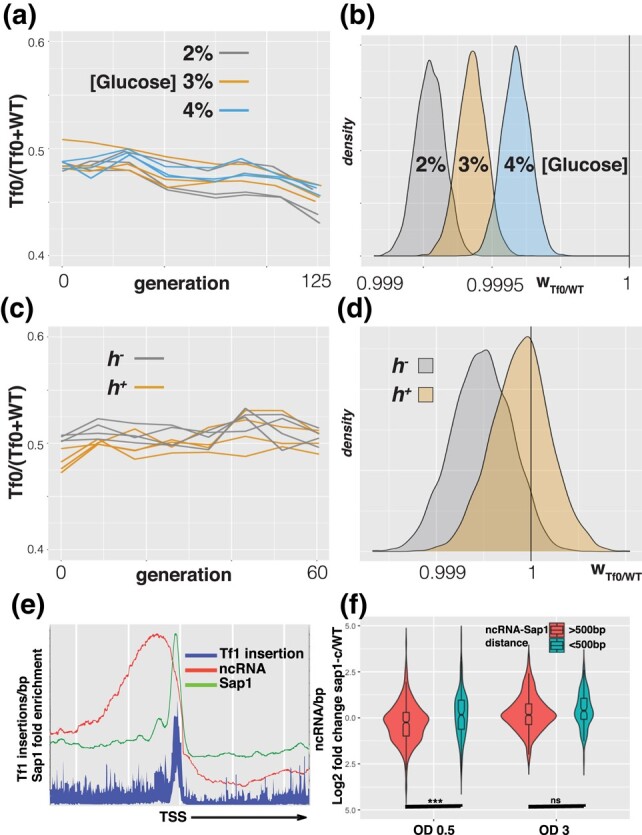
a) Frequency of the Tf-null specific polymorphism in competition cultures in increasing concentrations of glucose. b) Density plots of posterior probability distributions of relative fitness of Tf-null over WT (w_Tf0/WT_) from competition assays in (a). c) Frequency of the Tf-null specific polymorphism in competition cultures carried to saturation between each passage. d) Density plots of posterior probability distributions of relative fitness of Tf-null over WT (w_Tf0/WT_) from competition assays in (c). e) Distribution of Sap1 enrichment, Tf1 insertions, and ncRNA around protein coding gene Transcription Start Site. f) Log2 fold change of intergenic ncRNA expression by RNAseq, classified by their proximity to a Sap1 peak, as measured in early exponential growth (OD = 0.5) and early saturation growth (OD = 3). (***P < 0.001; ns: *P* > 0.05).

If the Tf2 insertions in the WT strain confer a competitive advantage over the Tf-null strain by modifying the regulation of metabolic genes, we would expect that the relative fitness would be sensitive to growth conditions. To evaluate this hypothesis we performed competitive growth assays in different growth media and passaging regimes. Changing the glucose concentration in the media from 2% to 4% revealed that the relative fitness of the Tf-null strain changes gradually, becoming more neutral with increasing glucose concentrations (1.6e−4 g^−1^; [9.8e−5, 2.1e−4]: β_glucose_ median; [HPDI 89%]) (Fig. 5a and b). A competition assay performed allowing the cultures to reach stationary phase passaging them every 2 days rendered the Tf2 insertions completely neutral, with w_tf0/wt_ = 0.9999; [0.9994, 1.0004] (median, [HPDI 89%]) (Fig. 5c and d). These results indicate that the effect of the Tf2 insertions on host fitness depends on growth conditions, further implicating the regulation of metabolic genes.

The influence of Tf2 insertions on host fitness through regulation of metabolic genes raises the possibility that the TE has evolved targeting strategies geared toward rewiring ncRNA dependent *cis*-regulatory cassettes. Tf element insertion is guided by Sap1 (Hickey et al. 2015; Jacobs et al. 2015) (Fig. 5e). *sap1* mutants exhibit a precipitous loss of viability upon reaching the stationary phase in rich media (Jorgensen et al. 2018), suggesting that its function is important for the response to metabolic stress. We evaluated whether Sap1 is involved in the regulation of intergenic ncRNA by RNAseq of the *sap1-c* mutant growing in early exponential (OD = 0.5) and early saturation (OD = 3) phases in rich media. This analysis revealed that the *sap1-c* mutant in exponential growth shows upregulation of ncRNA located near Sap1 binding peaks (Fig. 5f) and genes involved in the Core Environmental Stress Response (supplementary table S2, Supplementary Material online). These results indicate that Sap1 directly represses ncRNA expression during exponential growth. Taken together, these results suggest that Tf1 and Tf2 target ncRNA regulatory networks that respond to changing metabolic needs.

## Discussion

Using a laboratory type strain with all Tf2 removed, we can observe that the set of Tf2 insertions does provide a net fitness advantage to its host. While the fitness effect is relatively strong once the effective population size of fission yeast (Brown et al. 2011; Farlow et al. 2015) is considered (N_e_s ∼ 2e4), it is also highly dependent on growth conditions, being effectively neutral upon growth to saturation. This could indicate that the fitness effects of insertions present in a natural isolate are transient and highly dependent on changing environmental conditions. As a result, surveys of Tf colonies in natural isolates might show no signs of positive selection, reflecting instead recent transposition activity and population structure (Bourgeois and Boissinot 2019; Tusso et al. 2022).

The insertional preference of Tf1 and Tf2 for the promoters of protein-coding genes provides a potential mechanism for the fitness contribution of TE insertion. Tf1 can provide an enhancer activity increasing the expression of nearby genes (Feng et al. 2013; Esnault et al. 2019). However, this activity is not universal, as not all promoter insertions result in transcriptional changes. Consistently, in our analysis of all the Tf2 insertions present in the type strain, we could only observe changes in the expression of genes located next to the Tf2-7/8 array. The original insertion that later expanded to an array would have separated the *mic10* gene, involved in respiratory metabolism, from 2 ncRNA likely driven by the Scr1/Tup11/Rst2 transcription factors that orchestrate transcriptional responses to changing carbon source conditions (Vassiliadis et al. 2019). It is possible that the Tf2-7/8 array is the only insertion providing a fitness benefit to the laboratory-type strain, and that its tandem structure is the result of selection acting on the outcome of recombination on this insertion.

The fact that the positive fitness effect of the Tf2 insertions fades when growth is driven to saturation (Fig. 5c and d) supports the potential involvement of the metabolic needs of the host. Fission yeast natural isolates are closely associated with human activities involving fermentation (Jeffares 2018), where an initially glucose-rich environment supports rapid growth until glucose is exhausted and other carbon sources must be used (Malecki et al. 2016). In fission yeast, the transcriptional regulatory changes that accompany this change, termed the diauxic shift, are often carried out by ncRNA that control protein-coding genes in *cis* (Oda et al. 2015; Takemata et al. 2016). We have shown that Sap1, the DNA binding factor that guides Tf1 and Tf2 elements to insert on type II promoters, regulates ncRNA expression near genes associated with the core stress response pathways. Tf1 and Tf2 could alter the regulation of genes involved in the diauxic shift by severing their association with *cis*-regulatory ncRNA (Mastrangelo et al. 1992). Indeed, the Sap1 binding region present in the Nucleosome Free Region of protein-coding genes is ideally placed to guide such mutations, as ncRNA are commonly located upstream of core promoters (Fig. 5e). It is worth noting that genes whose expression is affected by Tf1 insertion were very often ncRNA (Feng et al. 2013). While these insertional mutations may provide a positive fitness effect in specific growth conditions, they nevertheless constitute a loss of complexity that can come with evolutionary tradeoffs. The regulation of the diauxic shift is itself an evolvable trait that can drive the evolution of carbon source generalist and specialist variants, but any such commitments are made at the expense of fitness in some conditions (Chu and Barnes 2016). Since ncRNA shows no coding potential the change induced by Tf1 or Tf2 insertion may be completely or partially reversible through deletion by ICR (Mastrangelo et al. 1992). In this manner, the regulation of metabolic and stress response genes may be rapidly and dynamically altered by TE element activity responding to changing environmental conditions. Traces of repeated insertion and deletion are visible in the fission yeast genome, where some gene promoters exhibit multiple LTR remnants of ancestral insertions, and independent insertions into the same promoters, sometimes in the exact same position, are observable in genomes of natural isolates (Jeffares et al. 2015; Tusso et al. 2022). Early studies of the influence of Ty1 elements on *S. cerevisiae* experimental evolution showed that, while fitness-negative on average, some insertions could become predominant by providing adaptive changes (Wilke and Adams 1992). Theoretical models show that fluctuating environments can enable the persistence of stable TE colonies in asexual and selfing populations if TE provides fitness or evolvability benefits to the host (Startek et al. 2013; Park et al. 2021). We speculate that the persistence of Tf elements in fission yeast genomes might depend on their dynamic contribution to fitness.

A recent study that deleted each individual Tf2 insertion by substitution with an antibiotic resistance expression cassette revealed no fitness effects, suggesting that the insertions present in the type strain are largely neutral. These results are not incompatible with the positive fitness effect we present here. First, the competitive growth studies carried out by Wang et al. were performed by growth to saturation, which as we have shown negates the fitness effect of the Tf2 insertions. Secondly, if the mechanism of fitness influence is, as we propose, the disruption of *cis*-acting regulatory ncRNA, substituting the Tf2 insertion with a different protein-coding gene expression cassette would leave this disruption unchanged, resulting in no changes to fitness. We believe that the removal of the Tf2 insertion by ICR, which leaves a solo LTR with no coding potential behind, is a more biologically relevant deletion intervention, as it is the main mechanism of LTR retrotransposon deletion in all organisms. Nevertheless, since we have only assayed the effect of all insertions as a set and not individually, we can only conclude that one or more insertions have a positive fitness effect. It remains possible that the fitness effect of individual insertions ranges from positive to negative, with a positive net aggregate in the assayed growth conditions.

While Tf2 insertions can provide a fitness advantage to its host, the negative effects of CoCl_2_ treatment and mating type on the WT strain indicate that they may also constitute a genetic burden that could offset their positive effects. Both CoCl_2_ treatment (Esnault et al. 2019) and Mc expression (Matsuda et al. 2011) may drive increased Tf2 transcription that could result in replicative stress (Hedges and Deininger 2007; Cam et al. 2008; Zaratiegui et al. 2011). An experiment in *S. cerevisiae* that overdosed its genome with Ty1 copies revealed that they cause a decreased capacity to survive genotoxic insults and inhibitors of DNA replication (Scheifele et al. 2009). A similar mechanism could explain the negative fitness effect of Tf2 transcription increase. The growth rate of natural isolates upon challenge with CoCl_2_ (Jeffares et al. 2015) is negatively correlated with TE copy number (supplementary fig. S4, Supplementary Material online), supporting a model where overall Tf1 and Tf2 dosage decreases fitness when TE expression is induced.

Tf2 insertions may also affect fitness by perturbing genome organization. Notably, Tf2 sequences cluster within the nucleus and form the Tf body, by way of protein factors that bind to the LTR (Cam et al. 2008; Tanaka et al. 2012). If the 2 LTRs flanking a Tf2 interact together this could prevent interaction with other dispersed LTRs, buffering their influence on nuclear architecture. The loss of the Tf2 CDS together with one of the LTRs might result in changes in genome 3D organization. The Tf-null strains can be used to evaluate this effect.

In summary, we have shown that the Tf2 insertions present in the fission yeast-type strain provide a fitness benefit. We speculate that the involvement of Sap1, the main driver of Tf1 and Tf2 insertion site preference, in the regulation of ncRNA expression in response to growth conditions could indicate that these TE have evolved a target site selection mechanism directed at manipulating the host's response to changing metabolic needs. The resulting fitness effect is small in comparison to other that of polymorphisms present in the global fission yeast gene pool. As an example, a low-activity Pyruvate kinase (Pyk1) variant found in the laboratory strain and a few related isolates exhibit a selection coefficient of s = 0.047, over 20-fold larger in magnitude than that attributable to the combined Tf2 insertions (Kamrad et al. 2020). But from the perspective of Tf2, which has very low transposition rates (Sehgal et al. 2007; Murton et al. 2016), it might mean the difference between persistence and extinction. In order to fully describe the relationship between Tf1/2 and its fission yeast hosts it will be necessary to measure the parameters driving their copy number dynamics (transposition and deletion rates) in natural populations and in growth conditions closely resembling the native fission yeast environment (Brysch-Herzberg et al. 2022). At this point, we can only speculate as to the role of Tf2 insertion site preferences and the effect of gene regulation at the inserted locus on the evolution of their host genomes. More experiments will be necessary to define the effect of individual insertions in different growth environments. The availability of a Tf-null strain will assist in these efforts by enabling experiments to directly test relevant hypotheses. More generally, we show that these and other fundamental aspects of TE biology (Wilke and Adams 1992; Ahn et al. 2017; Li et al. 2022) may be directly addressed through the use of transposon-free strains.

## Materials and Methods

### Analysis of Natural Isolates Sequence Data

To re-analyze the sequencing data from Jeffares et al. (2015) we downloaded the raw FASTQ files corresponding to the nonredundant 57 clonal strains identified in this study from the European Nucleotide Archive Accession PRJEB2126. We carried out copy number analysis with the DeviaTE pipeline (Weilguny and Kofler 2019) on trimmed, quality filtered, and deduplicated reads using a database including the divergent region of the gag gene from Tf1 and Tf2 (positions 56 to 1,000 with position 1 being the first nucleotide after the 5′ LTR), and a set of single copy essential genes from regions showing no evidence of amplification or deletion (Jeffares et al. 2017) (myo2, mrpl24, cdc6, urb1, cog2, cca1, spc7, tti1, dna2, lsh1, rpc1, psm1, cut3, msi2, sfc3, cog1, med15, ero12, cdc7, rpb3, alp1, smc5, and sws2). The output files were parsed to retrieve the High Quality estimated copy number. To validate this approach, we plotted the DeviaTE HQ copy number estimates with the copy number detected by BLAST of the same region against long-read derived genome assembles from the strains for which these were available as reported by Tusso et al. (2019) (SRA accession PRJNA527756).

### Cloning and Constructs

All oligonucleotides used in this study are in supplementary table S3, Supplementary Material online. The plasmids for CRISPR-aided Tf2 removal were generated by ligation of phosphorylated and annealed oligonucleotide pairs containing the targeting sequence into CspCI digested pMZ374 (Jacobs et al. 2014). The plasmid for cys12 CRISPR/Cas9 mutation was generated through amplification of plasmid pMZ374 with primers oM2350/oM2351 and direct transformation into *E. coli* competent cells, producing plasmid pMZ907. The plasmid containing the *ura4* gene flanked by Tf2CDS homology regions (pMZ160) was generated by amplification of flanking homology regions of Tf2 from strain 972 genomic DNA with primer pairs Tfam_USF/Tfam_USR-ura4 and Tfam_DSF-ura4/Tfam_DSR. The fragments were then used in a megaprimer Polymerase Chain Reaction (PCR) reaction with plasmid pUR19, containing the *ura4* gene, and primers Tfam_USF/Tfam_DSR to generate a fragment that was then digested with XhoI and cloned into XhoI digested pUC19. For deletion of Tf2-fragment1 (which lacks the homology in the right arm of the fragment from pMZ160) we amplified the *ura4* gene with Tf2 homology arms from plasmid pMZ762 with primers oM1781/oM1225.

### Strains Growth and Genetic Manipulation

All strains used in this study are detailed in supplementary table S4, Supplementary Material online. The growth media used were: Edinburgh Minimal Media (EMM, US Biological E2205-1) with 3.5 g/L glutamate (EMMG) with dropout (DO) mix lacking the appropriate supplement (US Biological: DO-adenine D9175, DO-uracil D9535, DO-histidine D9520, DO-leucine D9525) for selection of auxotrophic markers; EMM with 5 g/L ammonium chloride (EMMN) for growth of prototrophic strains; Yeast extract 5 g/L with 3% glucose and 200 mg/L adenine (YEA) for nonselective growth; yeast nitrogen base (US Biological Y2030) 1.7 g/L with 5 g/L ammonium sulfate, 2% glucose, DO mix without uracil (US Biologicals D9535), 40 mg/L uracil and 1 g/L 5-fluoroorotic acid (US Biological F5050) (5-FOA) for selection of uracil prototrophs. All transformations were carried out using the lithium acetate/Polyethylene Glycol heat shock method.

### Generation of Tf-Null Strains

For CRISPR-aided Tf2 removal, we transformed strain PB1 with the plasmids detailed in supplementary fig. S1c, Supplementary Material online and plated in media lacking uracil (EMMG + DO-uracil). Survivors were genotyped for the presence or absence of each entopic Tf2 by colony PCR with combinations of oM1365 with one of oM1366-1377. Survivors with multiple Tf2 deletions were selected, streaked on 5-FOA media to remove the CRISPR plasmid, and retransformed with a different plasmid. The process was repeated until all Tf2 elements were deleted.

For *ura4*-aided Tf2 removal, strains CHP428 and CHP429 were transformed with XhoI digested pMZ160 and plated in media without uracil (EMMG + DO-uracil). Colonies were then genotyped for the position of the *ura4* gene by colony PCRs with primer oM1968 and one of the upstream primers specific for each entopic Tf2 copy (primers oM2033 to oM2044). Colonies with an unambiguously localized *ura4* insertion in an identified Tf2 entopic copy were grown to saturation in liquid YEA and spread onto 5-FOA media plates to select *ura4* loss events. The surviving colonies were genotyped for deletion of the Tf2 copy that received the *ura4* insertion by colony PCR with primer oM1365 and an upstream/downstream primer pair corresponding to the Tf2 copy of interest (downstream primers: oM1366-oM1377; Upstream primers: oM2033-oM2044). Candidates with deletions were genotyped for mating type with primers oM9/oM10/oM11 and for *ade6-M210/M216* allele with primers oM12/oM13 followed by XhoI digestion. Once classified in h− and h+ groups, the deletions were combined by crossing between mating-compatible strains and genotyped for segregation of the deleted allele as above. Segregants with the desired combinations were used for a subsequent cycle of Tf2 removal. In the process of removing all described Tf2 entopic copies we detected a new Tf2 insertion present in the CHP428/CHP429 background, located in coordinates I:4940032, which we termed Tf2-14 and deleted in the same manner as the rest. The complete genealogy of the *ura4*-aided Tf2 removal is detailed in supplementary fig. S2b, Supplementary Material online.

We performed long and short-read whole genome sequencing to fully characterize the genome of the original parental strains and the Tf-null derivatives. DNA was isolated from saturated cultures of each strain grown in YEA media with the Monarch gDNA isolation kit (New England Biolabs). For short-read illumina resequencing, the DNA was sonicated to an average size of ∼150 bp with a Covaris S220 focused ultrasonicator. Libraries were generated with the NEBnext Ultra II library generation kit (New England Biolabs) and sequenced in a MiSeq instrument. For long-read sequencing, libraries were generated with the Oxford Nanopore Technologies ligation sequencing kit and run in a Minion instrument with 9.4 flow cells.

The Tf-null strains ZB2950/ZB2952 and the parental strains CHP428/CHP429 were cured of the present auxotrophies (*ade6-M210* or *ade6-M216*, *leu1-32*, *his7-366*, and *ura4-D18*) to generate prototrophic strains by serial transformation with PCR fragments containing the WT alleles (primer pairs: oM12/oM13 – *ade6-M210*; oM2300/oM2301 – *leu1-32*; oM2302/oM2303 – *his7-366*, oM2304/oM2305 – *ura4*-*D18*) followed by selection in EMMG media without the corresponding supplement and confirmation by Sanger sequencing, resulting in strains ZB3152, ZB3153 (from CHP428 and CHP429, respectively) and ZB3154, ZB3155 (from ZB2952 and ZB2950, respectively).

The *cys12-A13S* mutation was recreated by cotransformation of pMZ907 and an homologous recombination donor generated by amplification with oligonucleotides oM2352/oM2353 from genomic DNA of strain ZB2950.

### Competitive Growth Assays

For competitive growth assays, pairs of otherwise isogenic h+ or h− Tf-null and WT prototrophic strains (ZB3152/ZB3154 and ZB3153/ZB3155) were grown separately in liquid EMMN to exponential phase, harvested and washed twice in EMMN, counted and combined in equal numbers to an Optical Density (OD) of 1, and then distributed to triplicate 5 mL cultures of EMMN or EMMN +250 μM CoCl_2_, or EMMN 2%, 3%, or 4% glucose at an OD of 0.025. 1 OD of the initial mix was harvested by centrifugation and frozen as Generation 0. The cultures were serially transferred to 5 mL of fresh media twice a day to OD 0.1 in the morning and OD 0.025 at night, to prevent them from growing past OD 3. We measured OD at every passage to keep a record of the generations passed. Every 2 days ∼1.5 OD of each culture was harvested by centrifugation and frozen. After 8 timepoints had been harvested (14 days, mean = 136.4 generations and Standard Deviation (SD) = 0.55 for EMMN 2% to 4% Glucose, mean = 124.8 generations and SD = 0.52 for EMMN + 250 μM CoCl_2_) we stopped the experiment. We carried out the competition assay on saturation conditions similarly: pairs of h^+^ and h^−^ Tf-null and WT prototrophic strains were grown together as before, but cultures were passaged every 48 h to allow them to reach saturation: the cultures reached OD 10 within 24 h and remained at that OD until passage into fresh media to OD 0.025. We carried out the experiment for 14 d for a total of 60 generations.

To quantify the proportion of genotypes in the harvested competition cultures, we purified the genomic DNA from each timepoint with the Monarch gDNA extraction Kit (New England Biolabs). We then amplified the region of one of the mutations acquired by the Tf-null strain (I:1453108 C->A) with primers amenable to Amplicon-seq with in-line barcodes to identify the samples in pooled sequencing runs (oM2327-oM2339), purified the PCR products, measured their concentrations and pooled them in groups of 12 for Amplicon-Seq (Genewiz, Piscataway, NJ). Competitive growth assays to measure the fitness of the *cys12-A13S* mutation was carried out in EMMN-2% glucose maintaining passages at exponential phase.

We processed the resulting FASTQ files removing sequences shorter than 100nt, followed by splitting with fastx_barcode_splitter with options –bol –exact, and then trimming with fastx_trimmer -f 59 -l 63 -Q33 and then collapsing with fastx_collapser -Q33. The output files were parsed to separate the counts for the WT and Tf-null genotypes.

We analyzed the data fitting a Bayesian model specified in the STAN programming language in the R environment using package rstan. In this model^37^, the proportion of Tf-null genotypes in the competition:


pTf0=Tf0/(Tf0+WT)

progresses at each timepoint t, separated by Δg generations as:


$$\mathrm{pTf}0_{t}=\mathrm{pTf}0_{t-1}(w_{\mathrm{tf}0}+\beta_{\mathrm{mat}}\mathrm{mat}+\beta_{\mathrm{CoCl}2}\mathrm{CoC}l_{2}{)}^{\Delta g}$$

where w_tf0_ is the relative fitness of the Tf-null/h + strain in EMMN media with respect to the WT/h + strain, mat and CoCl_2_ are 0|1 variables representing the h^−^ genotype and CoCl_2_ 250 μM treatment, β_CoCl2_ and β_mat_ are coefficients representing the effect of CoCl_2_ treatment and h^−^ genotype. Similarly, the influence of Glucose concentration was estimated as a coefficient β_gluc_ representing the change in w_tf0_ per gram/100ml of additional glucose over 2% in the media. When comparing the relative fitness of the Tf-null strains with that of the Cys12A13S mutants obtained by CRISPR, the influence of lacking the Tf2 insertions was estimated as a coefficient β_Tf-null_ representing the change in w in the Tf-null strain. Relative fitness w_tf0_ was considered consistent with the null hypothesis (no difference between fitness of Tf0 strain over WT) if the Highest Posterior Density Interval (HPDI) at the 89% level of probability for the parameter included the value of 1, and the β coefficients were considered consistent with the null hypothesis (no influence of the estimated factor on w_tf0_) if the HPDI included the value of 0.

### Gene Expression Analysis

To study gene expression differences between the WT and Tf-null strains we grew triplicate 50 mL EMMN cultures of ZB3153 and ZB3155 in the presence and absence of CoCl_2_ 250 μM from an OD 0.025 to OD 1. We then harvested the cultures, washed them in ice cold water, and snap froze the pellets in a dry ice/ethanol bath. We purified total RNA with hot acid phenol extraction in TE +1% Sodium Dodecyl Sulfate, followed by 2 phenol:Isoamyl alcohol extractions and 1 Chloroform:Isoamyl alcohol extraction and precipitation with 250 mM NaCl and 3 volumes of Ethanol. We air-dried the pellets and resuspended them in Diethyl Pyrocarbonate-treated water. We treated 100 μg of total RNA with the DNAfree DNA removal system (ThermoFisher) and submitted for strand-specific total RNAseq to Genewiz (Piscataway, NJ). For analysis of ncRNA expression in WT (972) and *sap1-c* (ZB973) strains, we seeded from EMMN cultures grown overnight into YEA at OD 0.025 and harvested the cultures at OD 0.5 (early exponential growth) and OD 3 (early saturation). RNA was extracted and processed as above.

The FASTQ reads were filtered and trimmed with Trimmomatic 0.32, mapped to the gtf annotation file of the EMSEMBL genomes ASM294v2 assembly with TopHat 2.1.1 with options -r 200 –library-type fr-firststrand, followed by the HTSeq v1.12.4 framework HTSeq.scripts.count -s reverse to assign counts to features. The count data of the CoCl_2_/Tf-null experiment was analyzed with R package DEseq2. The expression of intergenic ncRNA (annotation retrieved from pombase.org/query with product type: feature_type_ncRNA_gene and then filtered to retain only intergenic ncRNA) in the *sap1-c*/growth stage experiment was analyzed with CuffLinks/CuffDiff and R packages lmer4 and emmeans. ncRNA were classified as Sap1-associated if they were within 500 bp of a significant Sap1 summit as assessed by Model Based Analysis of ChIp-Seq (MACS) analysis. Functional enrichment analysis was carried out on protein-coding genes from the EMSEMBL genomes ASM294v2 assembly annotation as assigned by closest proximity to Sap1-associated ncRNA, using the AnGeLi (Bitton et al. 2015) Web Interface (http://bahlerweb.cs.ucl.ac.uk/cgi-bin/GLA/GLA_input).

## Supplementary Material

Supplementary material is available at *Genome Biology and Evolution* online.

## Supplementary Material

evae010_Supplementary_Data

## Data Availability

All High Throughput Sequencing data generated in this work are available in the National Center for Biotechnology Information/NIH Sequence Read Archive under Bioproject ID PRJNA767600.
