# Supplementary material for: Transposon Removal Reveals Their Adaptive Fitness Contribution
Source: Genome Biol Evol. 2024 Jan 20;16(2):evae010. doi: 10.1093/gbe/evae010 (PMC10836971; doi:10.1093/gbe/evae010)
Supplement: evae010_Supplementary_Data [file evae010_supplementary_data.zip › Cranz_Mileva_Supplementary _data.pdf]

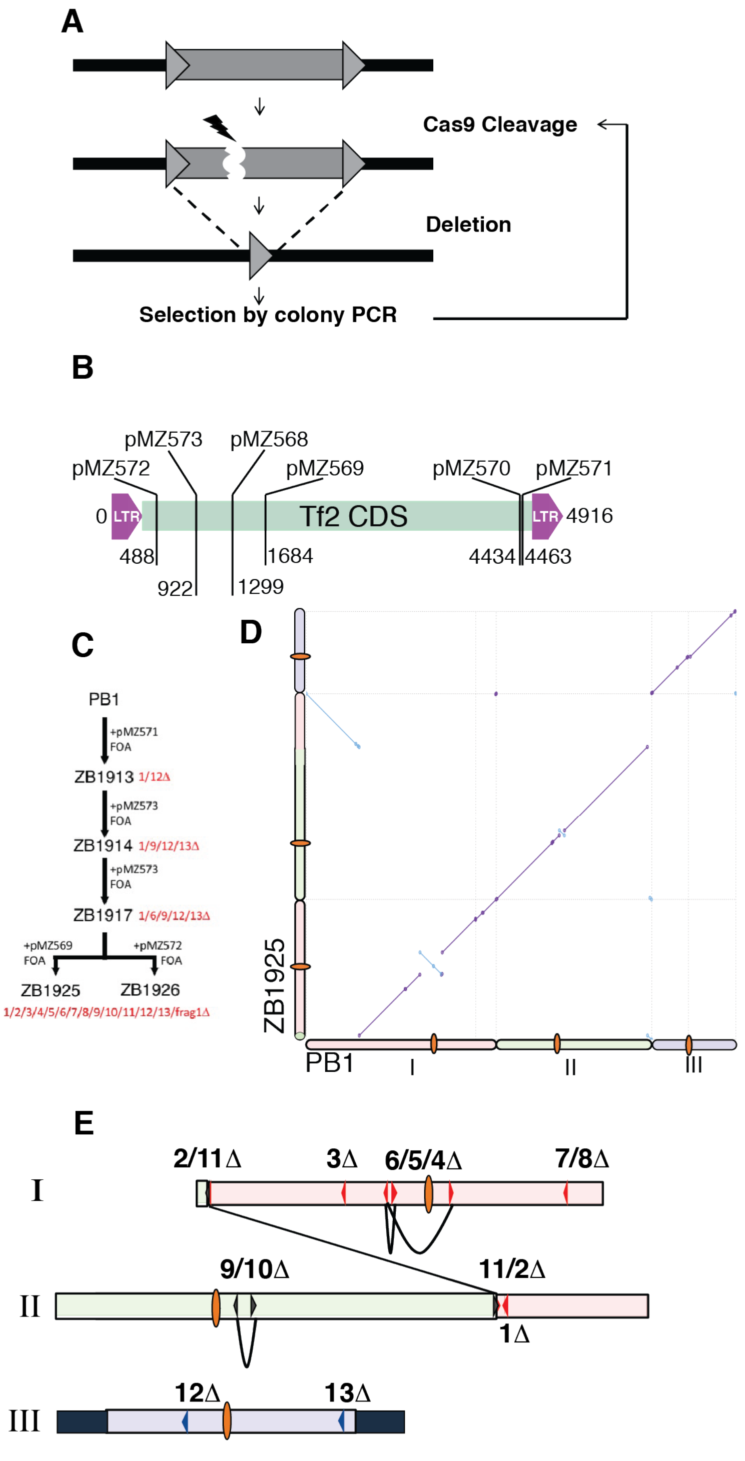

Supplementary Figure 1: CRISPR aided Tf2 deletion causes widespread rearrangements. a. Deletion strategy. b. Sites targeted by gRNAs, with plasmid numbers. c. Strain numbers and transformations followed, with Tf2 genotypes in red. d. Whole genome alignment of a CRISPR-derived Tf-null strain (ZB1925) with the parental strain (PB1)

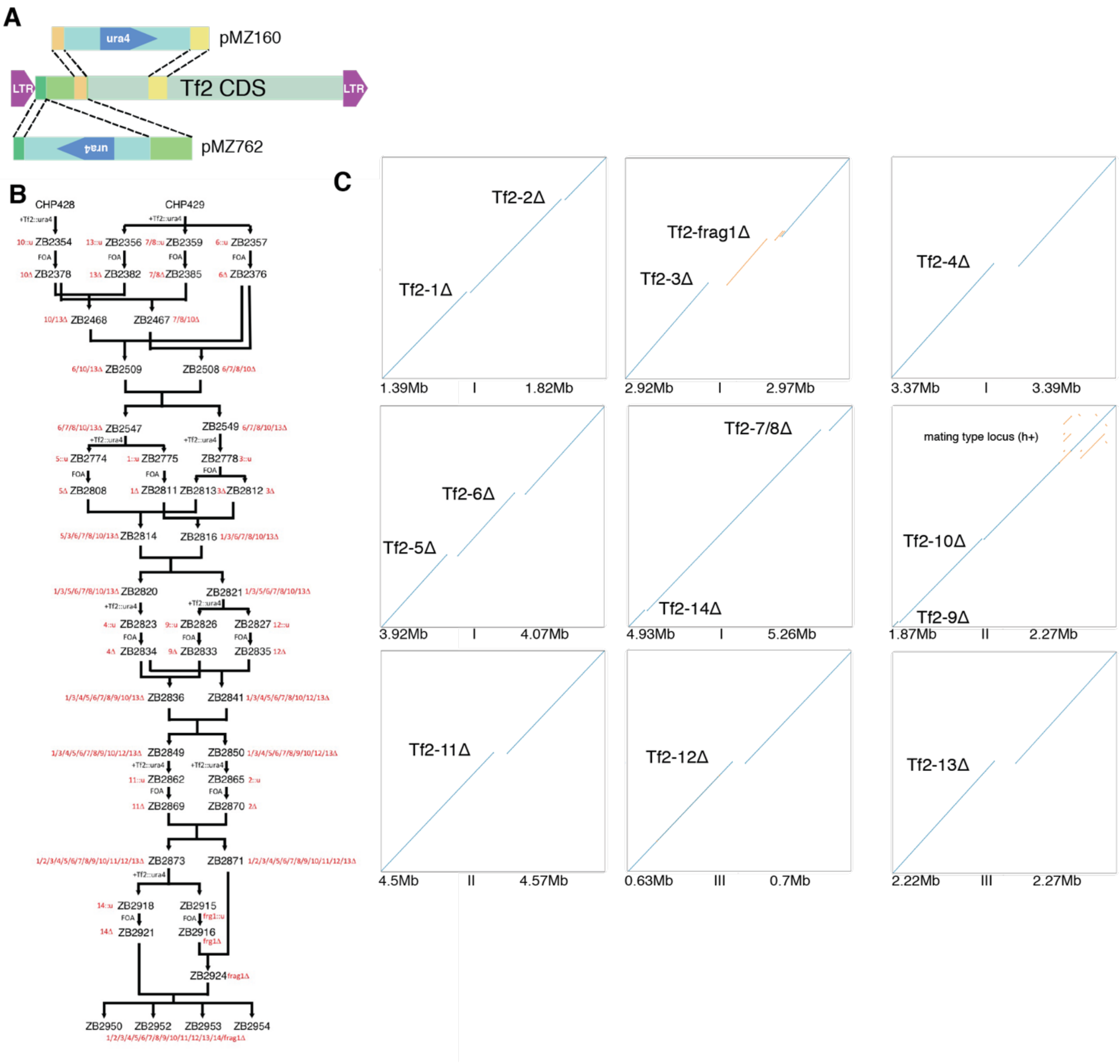

Supplementary Figure 2: 5FOA-Counterselection aided Tf2 deletion. a. Map of Tf2:ura4 targeting constructs. b. Strain numbers, transformations and crosses followed, with Tf2 genotypes in red. c. Whole genome alignment between a Tf-null strain (ZB2950, y axis) and a parental strain (CHP429, x axis) showing the deletion of all Tf2 elements.

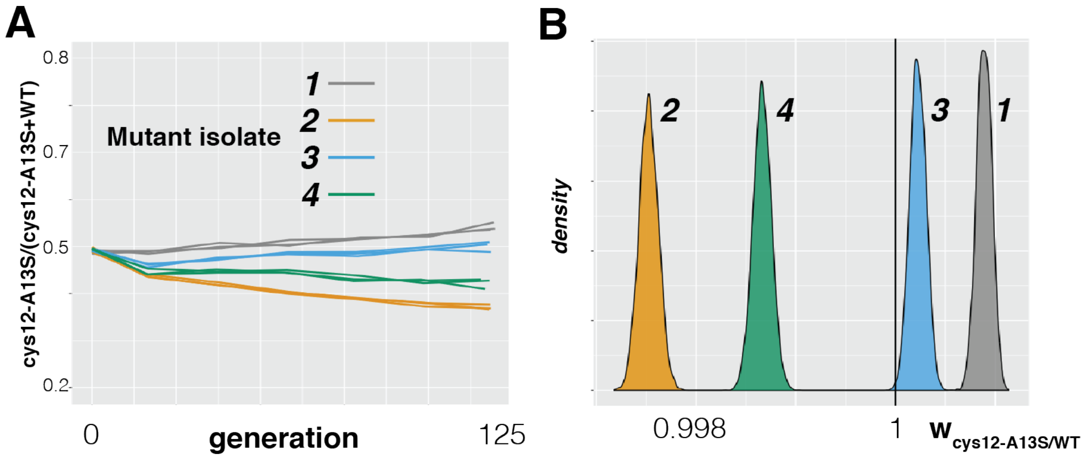

Supplementary Figure 3. WT versus *cys12-A13S* mutants competitive growth assays. a. Frequency of the *cys12-A13S* mutation in competition cultures of 4 independent mutant isolates b. Density plots of posterior probability distributions of relative fitness of *cys12-A13S* over WT ($w_{cys12\text{-}A13S\ /\mathrm{WT}}$).

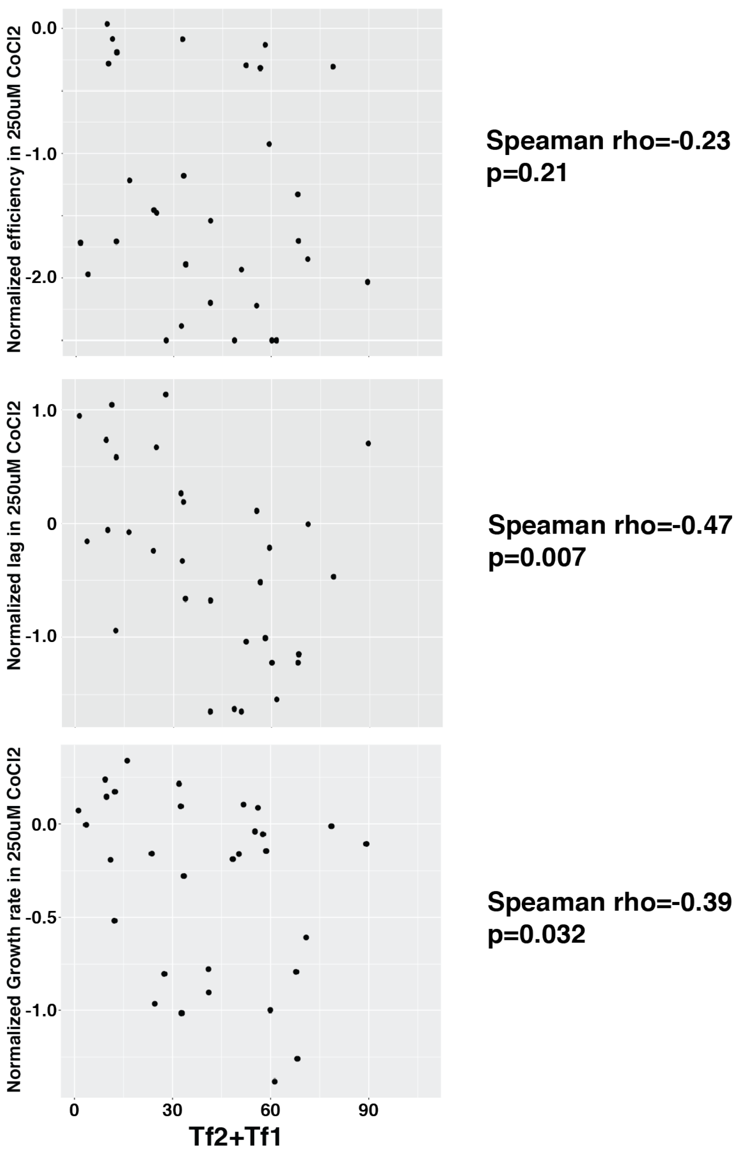

Supplementary Figure 4. Scatterplot of growth parameters in the presence of $CoCl_2$ of natural isolates described in Brown et al 2011.
